# Game Faces: How Digital Play Affects the Psychological Landscape of Youth

**DOI:** 10.7759/cureus.77497

**Published:** 2025-01-15

**Authors:** Kyndle S Lager, German Corso

**Affiliations:** 1 Psychiatry, Saint James School of Medicine, The Valley, AIA; 2 Child & Adolescent Psychiatry, Tropical Texas Behavioral Health, Harlingen, USA; 3 Pediatric Psychiatry, Tropical Texas Behavioral Health, Harlingen, USA

**Keywords:** child and adolescent psychiatry, child and youth mental health, ocd/ anxiety disorders, oppositional defiant disorder, suicide and depression, video game

## Abstract

The use of video games in children and adolescents has been growing since the invention of home interactive entertainment. With that growth, many parents and mental health professionals alike have questioned the impact on the mental well-being of their children and patients. Using current literature, we shall investigate the impact of video gaming on children's and teenagers' mental health in this systemic review. We will investigate time spent playing video games and the development of behavioral disorders, obsessive-compulsive disorder (OCD), depression, anxiety, and general psychological well-being. A search of PubMed and EBSCO discovery host was done, looking for primary peer-reviewed articles on the mental health outcomes of video gaming in the pediatric population (2-18 years old) of North America with no prior mental health diagnosis. The search returned 713 articles. After screening and selection, nine articles on six distinct studies were included. Overall, increased time spent playing video games was linked to increased depression and OCD symptoms, behavioral disorders, and suicidal ideation. This is a multifactorial issue that lacks substantial research in the current literature, leaving an opportunity for expansion on this topic in the future.

## Introduction and background

From the Nintendo 64 to the Play Station 5, home interactive entertainment has captivated children and adolescents since its conception. According to multiple studies, 90% of teenagers use video games, and they play video games for an average of 11.3 to 13.2 hours a week, suggesting that the prevalence of video gamers among young people may even be higher [1]. Many healthcare professionals have questioned the repercussions of this video game surge on the health of children and adolescents.

As home gaming consoles and personal computers have become more accessible, the age of initial exposure to video games and the Internet has gotten younger. Three-quarters of American families own a video game system and over 90% of children older than two play video games [1]. According to López‑Bueno et al., in a study of adolescents from 52 countries, adolescents who reported using the Internet for the first time at the age of 13 or older had the lowest probability of using it heavily (> 2 hours per day) when compared to those who started using it earlier (≤ 9 years) [2]. When considering the effects of video games on children and adolescents, it is important to consider age in introducing these games and the Internet. Other factors play a role in the effects of video games on children's lives, like how they affect activity levels and sleep.

Except for Just Dance, Ring Fit Adventure, and others that require physical activity, most video gaming perpetrates a sedentary lifestyle. Sleep duration in adulthood is correlated with being sedentary throughout adolescence. While playing video games, particularly those with violent themes, is associated with harmful effects on sleep, sedentary behavior during adolescence may have long-term effects on the amount of sleep an adult gets [3]. In contrast, physical activity during adolescence is not significantly related to sleep duration in adulthood [3].

Another lingering question with regard to video gaming and children is its effects on mental health. In this review, we will try to find out how video gaming affects the mental health of children and adolescents in North America. We will look at the effects on depression, anxiety, obsessive-compulsive disorder (OCD), behavioral disorders, and overall psychological well-being.

## Review

Methods

For this systemic review, we aimed to examine all studies available on the effects of video games on mental health in children and teenagers. To conduct a review of the literature, we used Pubmed and EBSCO Discovery Service. The search was restricted to work released between the years 2020 and 2024. To ensure a thorough search, a combination of keywords was used: ("video game*" OR "computer game*" OR "console game*" OR "mobile game*" OR "action game*" OR "simulation game*") AND ("mental health" OR "psychological well-being" OR anxiety OR depression OR "self-esteem" OR aggression OR behavior* OR cognition OR attention OR "executive function") AND (child* OR adolescent* OR youth OR pediatric OR "young people") AND ("United States" OR America OR US OR "United States of America") AND (2020:2024[PDAT] AND English[lang] AND peer-reviewed[journal]).

Only studies centered on children and teenagers from 2 to 18 years old that involved research about the effects of video games on psychological well-being were picked. We excluded studies that focused on the exacerbation of pre-existing mental health disorders. Our aim was to focus on newly developed diagnoses or harm to mental well-being due to playing video games. Acceptable research studies took different forms, including associated intervention studies, prospective cohort studies, retrospective cohort studies, and study designs where the dependent variable was measured only once (cross-sectional). Only articles specifically reporting on mental health outcomes were included to ensure relevance. Studies that did not meet these criteria, such as those involving non-peer-reviewed publications, non-English language articles, or research conducted outside the United States, were excluded. Review articles, meta-analyses, editorials, commentaries, and case reports were excluded. The "Excel Workbook for Screening Titles and Abstracts" was used to screen abstracts and titles [4].

For each study that met the inclusion criteria, data were extracted regarding the study's characteristics such as author(s), year of publication, study design, sample size, population characteristics, duration and frequency of intervention or exposure, and the presence of a control group. Mental health outcomes, the instruments and techniques used to evaluate them, and specific findings found were further outlined. The method of confirming the study's validity involved qualitative techniques to extract and critique certain elements such as sample size, study design, and SD components. A risk-of-bias assessment was done using the Critical Appraisal Skills Programme (CASP) Qualitative Checklist [5]. This tool evaluates various domains, such as clarity of research aims, appropriateness of the methodology, data collection processes, and ethical considerations, to identify potential sources of bias in qualitative studies.

Results

**Figure 1:**
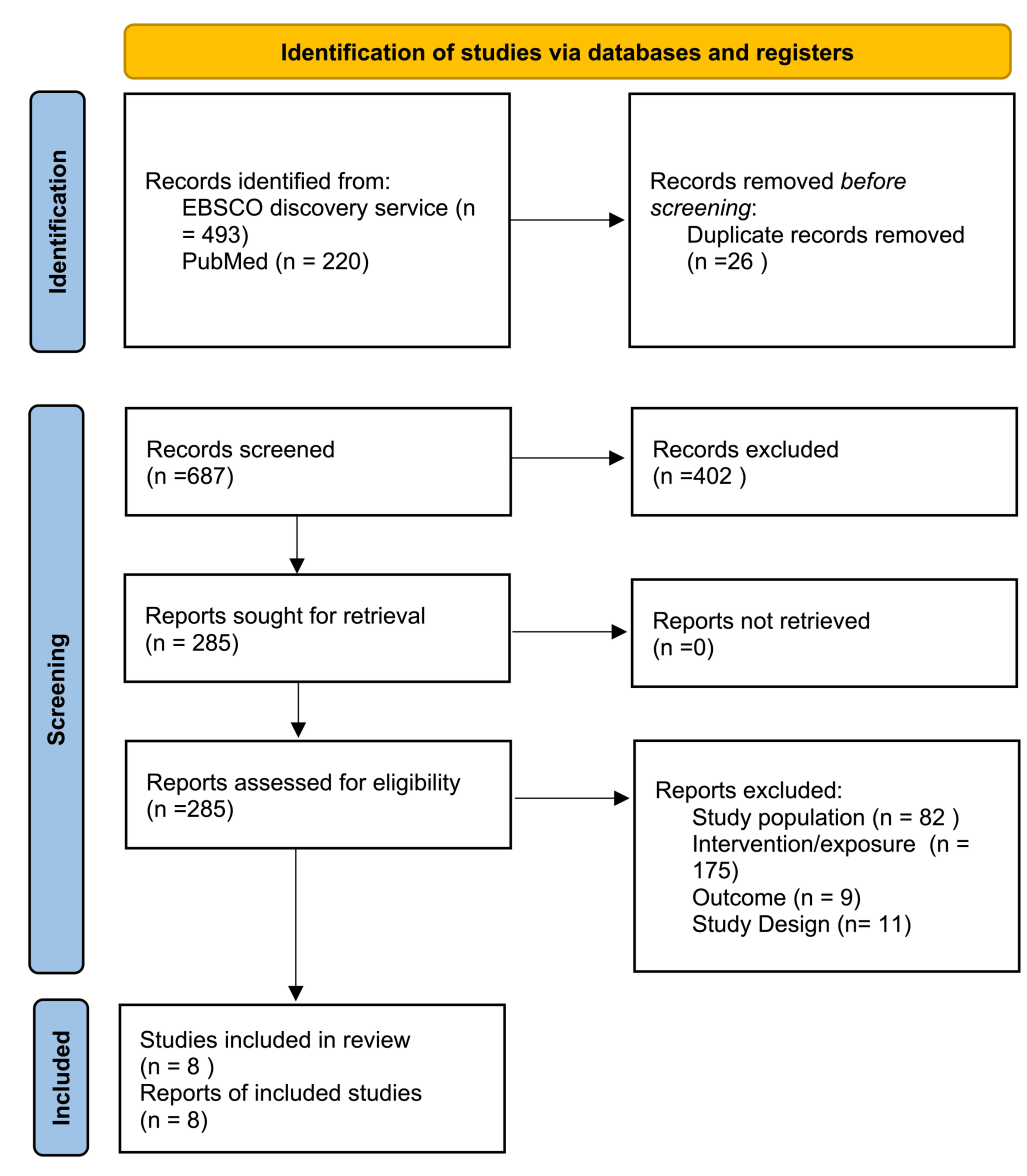
PRISMA flow diagram PRISMA: Preferred Reporting Items for Systematic reviews and Meta-Analyses Source: [6]

A total of 713 articles were returned upon initial search, 493 from the EBSCO Discovery Service and 220 from PubMed. Of these, 26 articles were removed before screening due to duplication. That left 687 articles to be screened, of which 402 were excluded. A total of 285 articles were successfully retrieved and assessed for eligibility. Exclusions included 82 articles due to population, 175 due to intervention/exposure, 9 due to outcome, and 11 due to study design. That left four studies, with eight reports of the included studies (Figure 1) [6].

**Figure 2:**
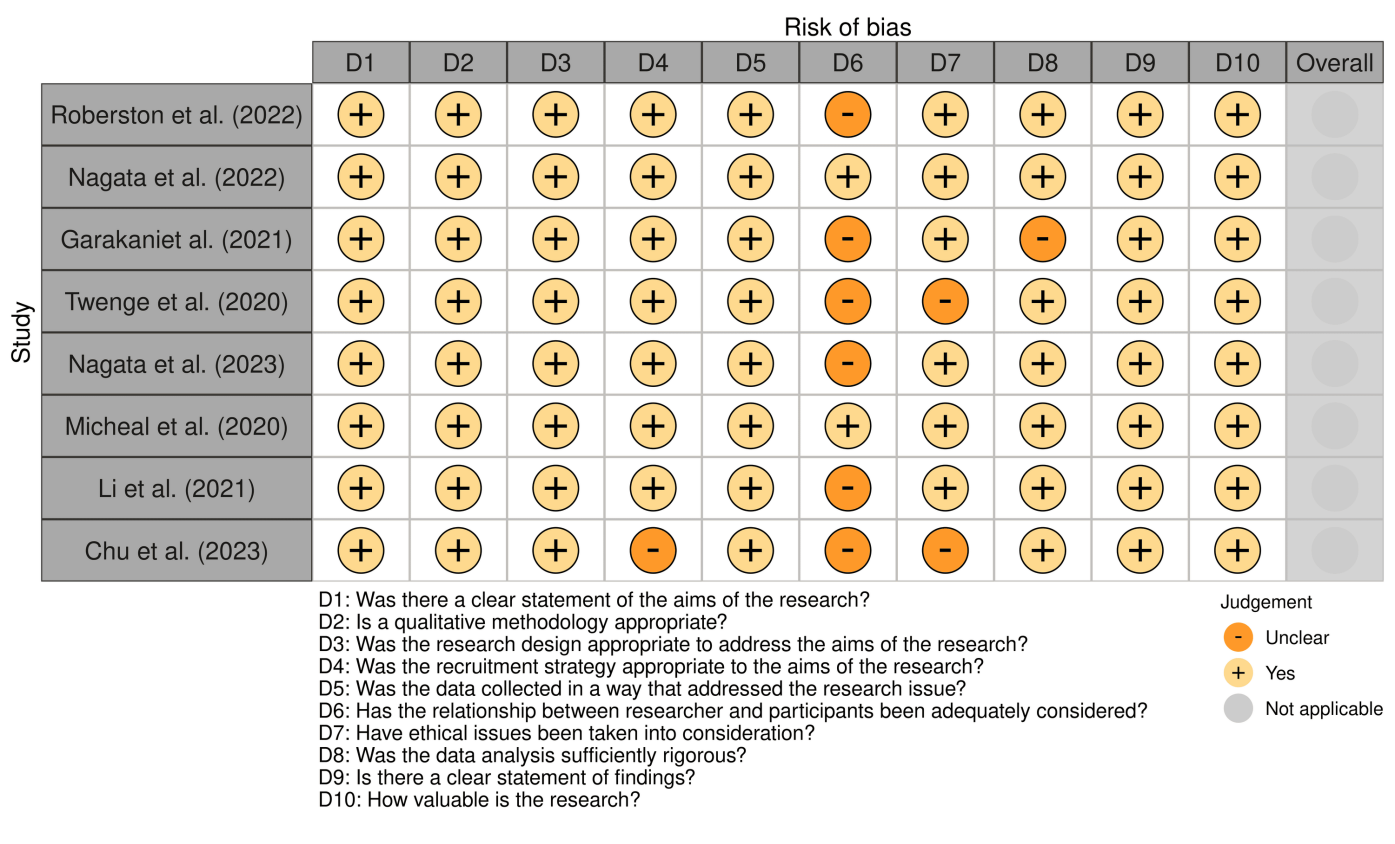
Individual qualitative studies' risk of bias (RoB), as determined by the Critical Appraisal Skills Programme's (CASP) Qualitative Checklist For every study, Domains 1–10 were assessed and answered as Yes (+), unclear (−), or No (+). Source: [7]

The risk-of-bias assessment (Figure 2) demonstrated a strong alignment between the studies’ research aims and the qualitative methodologies employed, with all studies using appropriate designs to address their research questions [7]. However, there were variations in the level of detail provided for certain aspects, such as researcher reflexivity and ethical considerations, which introduced some potential sources of bias. Every study used appropriate qualitative approaches and had well-defined research objectives. This uniformity implies that the studies were in a good position to address the research questions they were intended to address. While one study by Chu et al. [8] lacked comprehensive information regarding participant recruitment, raising possible concerns regarding representativeness, the other seven had sufficient recruitment procedures. How the researchers accounted for their interactions with participants was not sufficiently addressed in several studies (Robertson et al. [9], Garakani et al. [10], Twenge et al. [11], and Chu et al. [8]). Because the possible impact of the researchers' own biases on data collection and analysis was not completely taken into account, this could have an impact on how the findings are interpreted. Each study had a different level of ethical transparency. Some research (including Robertson et al. [9] and Twenge et al. [11]) did not explicitly state how confidentially was preserved or whether ethical approval was acquired, even though the majority of studies addressed ethical considerations. Most research had a thorough data analysis, while some, like Garakani et al. [10], only gave a limited amount of information about the analysis procedure, which might have impacted how in-depth the results were. In summary, some methodological constraints, especially with regard to researcher-participant reflexivity and ethical transparency, point to areas where potential bias could influence the results, even if the included studies generally showed sound qualitative procedures and clear goals. The interpretation of the review's findings took these limitations into consideration.

One multi-site longitudinal study, four cohort studies, and three cross-sectional studies were included. The population spanned from 2-year-olds to 18-year-olds. Four of the studies used the same Adolescent Brain Cognitive Development study data set; these include Chu et al. [8], Roberston et al. [9], Nagata et al. [12], and Nagata et al. [13]. The largest sample size is 200,000; the smallest is 2,005. You can also see a cluster in age, with a larger portion of the population being 9-10 years old (Table 1).

**Table 1:** Characteristics, results, and summaries of the included studies ARR = adjusted relative risk; CI = confidence interval; ODD = oppositional defiant disorder; CD = conduct disorder; GRTA = gaming to reduce tension or anxiety; OR = odds ratio; BMI = body mass index; OCD = obsessive-compulsive disorder; AOR = adjusted odds ratio; ABCD = Adolescent Brain and Cognitive Development Study; APR = adjusted prevalence ratio

Citation	Title	Data collection year (s)	Study Design	Sample Size	Population	Results	Summary
Roberston et al. [9]	Associations Between Screen Time and Internalizing Disorder Diagnoses Among 9- to 10-Year-Olds	2017-2018	Multi-site longitudinal study (used Adolescent Brain and Cognitive Development Study)	11,875	9-10-year olds	Girls who played games for more than 2 hours daily were more likely to have an anxiety disorder (ARR= 2.07; 95% CI= 1.12-3.84) and more likely to have Suicidal ideation or attempt (ARR= 1.23; 95% CI= 0.92-1.65). Boys that spent more than 2 hours gaming daily were more likely to have depressive disorders (ARR=1.39; 95%CI=1.11-1.76), Suicidal ideation or attempt (ARR=1.30; 95% CI= 1.10-1.53), and self-harm (ARR= 1.39; 95% CI= 1.14-1.70)	The researchers found that more time spent with a screen was associated with meeting the criteria for depressive disorders, anxiety disorders, self-harm, and thoughts of suicide or attempts. This study explored the association with internalized disorders and various forms of screen time which did show a different disruption of diagnoses. The authors note that causality cannot be claimed but due to preadolescents being such an important period for brain development, these associations warrant further investigation.
Nagata et al. [12]	Contemporary Screen Time Modalities and Disruptive Behavior Disorders In Children: a Prospective Cohort Study	2016-2019	Prospective cohort study (used Adolescent Brain and Cognitive Development Study)	11,875	9-10-year olds	Youth reported spending an average of 4.0(±3.2) hours a day on screens with 1.1(±1.1) spent playing video games. Baseline screen time spent playing video games showed a significant association with oppositional defiant disorder (ODD) at 1-year follow-up (Prevalence ratio = 1.14; 95% CI= 1.07-1.21; p<0.001). After adjusting no significant association was found between conduct disorder and time spent playing video games.	This article looked into the relationship between screen time and disruptive behavior disorders specifically conduct disorder (CD) and oppositional defiant disorder (ODD). The exposure was screen time, the outcome was CD or ODD. Based on previous literature the authors selected potential sociodemographic confounders were selected. Overall spending more than 4 hours daily on a screen significantly increased the prevalence of behavioral disorders at 1-year follow-up. The authors note that due to the nature of the study, a causal relationship cannot be determined. Additionally, self-reporting may cause an underestimation of time spent with screens. The strongest association that was found in this study was between social media use and CD so the authors encourage further research in this area.
Garakaniet al. [10]	Gaming To Relieve Tension or Anxiety and Associations With Health Functioning, Substance Use, and Physical Violence in High School Students	2021	Cross-sectional study	2005	High-school students in the 9^th^-12^th^ grades	Gaming to reduce tension or anxiety (GRTA) was found in 15.3% of participants. Boys were more likely to report GRTA than girls (χ2 =27.69; p < 0.001). GRTA was more likely in those who self-identified as Hispanic (χ2 =18.72; p <0.001) or Asian (χ2 =4.82; p =0.03). Adolescents with GRTA, based on adjusted multivariate analysis of Problematic Internet Use criteria, were more likely to report: family concern (OR=2.32; 95% CI=1.77-3.06; p<0.001), interference with school/work/activities (OR=7.49; 95% CI=5.23–10.73; p<0.001), recognizing they have a problem (OR=4.84; 95% CI=3.41–6.85; p<0.001), an irresistible urge (OR=5.99; 95% CI=4.45–8.08; p<0.001), and tension/anxiety (OR=14.35; 95% CI=10.26–20.07; p<0.001). GRTA is associated with an increased likelihood of feeling depressed or dysphoric (OR = 3.16; 95% CI = 2.33-4.28; p<0.001)	The study hypothesized that girls would report more gaming to reduce tension or anxiety (GRTA) than boys, and that GRTA would be linked to increased internet use, poorer academic performance, substance use, depressive symptoms, higher BMI, and greater involvement in physical violence and victimization. Findings revealed that GRTA was more common among boys and Hispanic/Latino adolescents, and was associated with problematic internet use, lower academic achievement, illicit drug use, smoking, depression, and violence. The authors suggest that treating underlying anxiety may impact depressive symptoms and recommend further research on the topic.
Twenge et al. [11]	Gender Differences in Associations Between Digital Media Use and Psychological Well-Being: Evidence From Three Large Datasets	2013-2016	Cross-sectional study	221,096	13- to 18-year-olds	Among US adolescents males spend an average of 10 minutes more a day gaming than females (p<0.001). Gaming for less than 30 minutes a day was positively correlated to well-being and happiness partially in males. With a minor effect size (d = 0.10), boys who played light electronic games reported higher levels of happiness than those who did not participate at all. However, compared to boys, girls only slightly benefited from light gaming, as seen by a smaller effect size of d = 0.02. This suggests that girls did not experience the same level of satisfaction from light gaming. When compared to light users, heavy gamers—those who played for five or more hours a day—were frequently twice as likely to report poor well-being. In particular, just 11% of boys who played for 30 minutes reported feeling psychological well-being, but 64% of boys who played for seven or more hours felt the same way. In a similar vein, girls who gamed for seven or more hours a day reported lower levels of well-being (73% against 15% for half an hour).	This study analyzed data from three large, nationally representative surveys of 13- to 18-year-olds in the U.S. and U.K. Findings show boys spend more time gaming than girls, particularly at school. Excessive gaming is linked to poorer well-being and mental health issues, with stronger negative effects observed in girls. Boys who engage in moderate gaming report slightly better well-being than non-gamers. The researchers recommend light gaming for boys to improve well-being and emphasize the need for interventions targeting heavy gaming, especially in girls. Further research is needed to examine the mental health impacts of gaming and the benefits of time restrictions.
Nagata et al. [13]	Screen Time and Obsessive-Compulsive Disorder Among Children 9-10 Years Old: A Prospective Cohort Study	2016-2020	Prospective cohort study (used Adolescent Brain and Cognitive Development Study)	9,208	9-10-year olds	When adjusted for covariates and excluding those with a diagnosis of obsessive-compulsive disorder (OCD) at baseline, increased total screen time (AOR=1.05; 95% CI=1.01-1.09; p<0.012), watching YouTube videos (AOR= 1.11; 95% CI=1.01-1.23;p<0.038) and video games (AOR=1.15; 95% CI=1.03-1.28; p<0.015) were associated with increased risk of an OCD diagnosis at 2-yeat follow-up. For video games in particular each additional hour spent playing (AOR=1.15; 95% CI=1.01-1.23) was associated with future OCD.	The objective of the research was to investigate the respective association of different types of screen exposure with the emergence of OCD in a cohort of 9-10-year-olds, who play a part in Adolescent Brain Cognitive Development (ABCD) research. The study was intended to fill in the voids in the literature specifically in relation to the effect of screen time on the development of OCD in this socio-cognitive critical age. Each extra hour of playing video games deepened the risk of developing obsessions over the period of two years, justifying a correlation between playing more video games and the appearance of obsessive-compulsive disorder in this group. It is recommended in the study that more comprehensive research is carried out to investigate the way video gaming contributes to the development of OCD in adolescents.
Micheal et al. [14]	Physical Activity, Sedentary, and Dietary Behaviors Associated with Indicators of Mental Health and Suicide Risk	2017	Cross-sectional study	14,765	High-school students in the 9^th^-12^th^ grades	Amongst female students who reported playing video or computer games or using a computer for more than 2 hours were more likely to feel sad and hopeless (APR=1.21; 95% CI= 1.09-1.34; p< 0.001) and had a serious consideration for attempting suicide (APR=1.42; 95% CI=1.25-1.63;p<0.001).In boys who reported playing video or computer games or using a computer for more than 2 hours they were more likely to have feelings of sadness and hopelessness (APR=1.30; 95% CI=1.13-1.50; p<0.001), had considered suicide (APR=1.58; 95% CI=1.32-1.90; p<0.001) and attempted suicide (APR=1.56; 95% CI=1.04-2.33; p<0.05).	This study examines the links between physical inactivity, sedentary behavior, poor diet, depression, hopelessness, suicidal ideation, and suicide attempts among American students. Among students spending over two hours on computers or gaming, suicidal ideation was reported by 58% of males and 42% of females. However, no significant relationship was found between suicide attempts and physical activity levels. Male students spending over two hours on screens were 56% more likely to attempt suicide. The authors recommend further research into the types of games and websites used by teens, as well as gender differences in media use. They suggest interventions to reduce prolonged screen time and sedentary behaviors while promoting physical activity and nutrition in schools to support mental health.
Li et al. [15]	Screen Use and Mental Health Symptoms in Canadian Children and Youth During the COVID-19 Pandemic	2020-2021	Cohort study	2026	2-18-year olds	Increasing hours spent playing video games was associated with increased depression symptoms (1 hour: β,0.74 (95%CI,−0.21- 1.69); 2-3 hours: β,2.54 (95%CI,1.15-3.57); 4-5 hours: β,4.30 (95%CI,2.94-5.66); 6-8hours: β, 6.28 (95%CI,4.46-8.09); 9 hours: β,5.17 (95%CI,2.69-7.64); overall p < .001).	The aim of this particular research was to delve into the relations between mental health disorder symptoms and distinct forms of screen usage among the children and youth of Canada within the sphere of the ongoing COVID-19 pandemic so that adequate healthcare policies and corrective measures can be designed, promoting the wholesome use of screens and robust mental health amongst this population. Excessive time spent playing video games contributed to mental health problems including depression, lack of focus, and overactive behavior and aggression in children and adolescents within the period that was most affected by the pandemic. Yet, the dimensions of this relationship are such that there are various degrees of regression, which suggests an uneven influence of playing video games on mental health. The research called for efforts in policy-making to implement norms and standards for the use of computers and mobile phones by children, which included but was not limited to, the return of children to schools, and prevention of excessive screen time, including that spent on video games.
Chu et al. [8]	Screen Time and Suicidal Behaviors Among U.S. Children 9–11 Years Old: A Prospective Cohort Study	2016-2020	Prospective cohort study (used Adolescent Brain and Cognitive Development Study)	11,633	9–11-year olds	When adjusted for covariates video game screen time was associated with suicidal behaviors at the 2-year follow-up (OR=1.18; 95% CI, 1.01-1.38; p<0.036).	This study aimed to ascertain the potential correlations between baseline screen use and suicidal behaviors in a large national cohort of 9–11-year-old American children two years later. According to the study, children aged 9 to 11 who play video games are more likely to engage in suicidal thoughts and behaviors. This suggests that playing video games more frequently may raise the risk of suicidal thoughts and behaviors. These results emphasize the necessity of more research on the relationship between video gaming and mental health as well as the significance of keeping an eye on teenage consumption.

Table 1 also includes the results and conclusions of the included studies. The first study by Roberston et al. [9] found that gaming for more than two hours daily (95% CI) was associated with anxiety disorder, adjusted relative risk (ARR) 2.07 (1.12, 3.84), and suicidal ideation (SI) or attempt, ARR 1.23 (0.92, 1.65), in girls. In boys, they found associations with depressive disorder, ARR 1.39 (1.11, 1.76), SI or attempt, ARR 1.31 (1.10, 1.56), and self-harm, ARR 1.39 (1.14, 1.70).

Nagata et al. reported that, at baseline, youth spent an average of 4.0 (±3.2) hours per day using screens, including 1.1 (±1.1) hours specifically playing video games [12]. Additionally, 38.7% of participants reported using screens for four or more hours daily. After controlling for confounders, adjusted regression analyses evaluating the prospective associations between baseline screen time and oppositional defiant disorder (ODD) at a one-year follow-up reveal that there was a prospective association between each hour of video game play and a higher prevalence of ODD (PR 1.14, 95% CI 1.07-1.21, p<0.05).

Next, Garakani et al. found that 319 of the 2005 respondents (15.3%) were found to have gaming to reduce tension or anxiety (GRTA) [10]. After stratifying the demographic characteristics of adolescents by GRTA status, a chi-square analysis showed that boys (χ2 =27.69; p < 0.001) and those who endorsed being Hispanic (χ2 =18.72; p <0.001) and Asian American (χ2 =4.82; p =0.03) were more likely to report GRTA. The Problematic Internet Use Criteria adjusted multivariate analysis stratified with GRTA showed that adolescents with GRTA were more likely to have: Family concern (OR= 2.32; 95% CI= 1.77-3.06; p<0.001), Interference with School, Work, Activities (OR= 7.49; 95% CI= 5.23-10.73, p< <0.00), Think that they have a problem (OR=4.84; 95% CI= 3.41-6.85; p <0.001), Irresistible urge (OR= 5.99; 95% CI =4.45-8.08; p<0.001), and Tension or anxiety (OR=14.35; 95% CI= 10.26-20.07, p<0.001). Adolescents who endorsed GRTA were more likely to have dysphoria or depression (OR= 3.16, 95% CI: 2.33-4.28, p< 0.001).

Twenge et al. stated that male adolescents in the United States play video games for an average of 10 minutes more per day than female adolescents (p<0.001) [11]. For males, gaming less than half an hour a day was positively associated with well-being. Boys who played light electronic games expressed greater levels of enjoyment than those who did not play any games at all, with a small effect size (d = 0.10). However, a smaller effect size of d = 0.02 indicates that girls only marginally benefited from light gaming in comparison to boys. Heavy gamers (those who played for five or more hours a day) were twice as likely to report low psychological well-being as light players. Specifically, 64% of boys who played for seven or more hours reported feeling low psychologically, compared to just 11% of boys who played for 30 minutes. Likewise, girls who played video games for seven or more hours a day expressed poorer levels of well-being (73% compared to 15% for 30 minutes).

According to Nagata et al., total screen time, watching videos, and playing video games were associated with OCD at a two-year follow-up [13]. Each additional hour of playing video games (OR 1.15, 95% CI 1.03e 1.28) was associated with subsequent OCD.

Furthermore, Micheal et al. discovered that female and male students who played video or computer games or used a computer for more than two hours per day were 42% and 58% more likely to have seriously considered attempting suicide, respectively [14]. Male students who played video or computer games or spent more than two hours per day on a computer were 56% more likely to attempt suicide than those who did not.

According to Li et al., during the COVID-19 pandemic, more hours of daily video game time were linked to higher levels of depressive symptoms in older children [15].

Finally, as explained by Chu et al., video game screen time was associated with increased suicidal behaviors (OR 1.18, 1.01-1.38, p < 0.036) [8].

Discussion

Studies have shown that spending long periods playing video games is associated with higher risks of depression, self-harm, and suicidal ideation in preadolescents. This underscores the importance of understanding how game content, immersive experiences, and online interactions impact mental health to create effective interventions and promote healthy gaming habits [8]. Moreover, adolescents who turn to gaming as a way to relieve tension or anxiety - more often boys and Hispanic and Asian adolescents - displayed lower participation in extracurricular activities, poorer academic outcomes, increased substance use, problematic Internet use, and higher rates of depression. Risky behaviors, such as carrying weapons, skipping school due to safety concerns, and physical altercations were also noted, suggesting a complex relationship that requires further research to tailor interventions [10]. Additionally, boys experienced a more vital link between light gaming and well-being, whereas heavy gaming correlated with lower well-being, especially in girls. These gender differences point to the importance of considering gender when examining the connection between gaming and psychological well-being in adolescents [11].

Each additional hour of video game use in children aged 9-10 was tied to a 15% higher risk of developing OCD within 2 years. These findings emphasize the need to recognize video gaming as a possible risk factor for OCD during early adolescence and call for more research and preventive measures [13]. In high school students, playing video games or using a computer for more than two hours per day was linked to feelings of sadness, hopelessness, and severe thoughts of suicide, and this prolonged screen time also correlated with greater social isolation and loneliness, with distinct differences based on sex. Understanding the role of social media and video game content may offer more insight into their varied impacts on mental health among male and female adolescents [14].

Furthermore, higher levels of video game use among older children and teens during the COVID-19 pandemic were associated with increased symptoms of depression, irritability, inattention, and hyperactivity. These connections were somewhat reduced in adjusted models, reflecting a more complex relationship between gaming and specific mental health symptoms. This suggests that monitoring and regulating video game usage could improve mental health outcomes in this population [15]. Finally, each additional hour spent playing video games was linked to a 1.18-fold increase in the odds of reporting suicidal behaviors among 9-11-year-olds at a 2-year follow-up. These findings highlight the potential link between video gaming and the increased risk of suicidal behaviors in this age group, emphasizing the need for parents, educators, and healthcare professionals to manage screen time and support children's mental well-being [8].

Almost all of the studies showed an increased prevalence of gaming amongst males vs. females. However, some studies showed worse mental health outcomes in females, and some showed worse outcomes in males. From this data, as time playing video games increases, so does the risk of developing depression, anxiety, OCD, or behavioral disorders. As well as increased risk of SI and self-harm. In the Gaming to Relieve Tension or Anxiety study [10], we saw decreased extracurricular activity engagement in children who used video games as a coping mechanism.

Interestingly, engaging in extracurriculars is linked to higher life satisfaction and optimism and lower anxiety and depression symptoms [16]. At the same time, over two hours of daily screen time is associated with worse mental health outcomes. Another interesting point of discussion is that joint media activities, like playing video games with siblings, have enhanced inhibitory control development in preschool children more than solitary media [17]. So, it might not be about what children are playing but if they are playing it with others or by themselves. Lastly, to touch on the multiple studies that showed increased depression symptoms with increased video game play, one study has shown that increased screen time can lead to anhedonia, which in turn raises the risk of substance use among adolescents, so video games may not only offer a gateway to a virtual world but may also be a gateway drug [18].

Limitations

The study's limitations include issues with generalizability, as four of the studies focused exclusively on 9-10-year-olds, making it difficult to apply the findings to broader age groups. Additionally, the relationship between gaming and mental health is complex, with challenges in determining whether problematic gaming leads to poor mental health or if pre-existing mental health issues drive increased gaming. Self-reporting bias is also a concern, as much of the data relies on participants' recollections of their gaming habits and mental health, which may be influenced by recall inaccuracies or social desirability. Moreover, while associations between gaming and mental health were observed, causality cannot be established, leaving uncertainty about whether gaming directly causes these outcomes. Finally, variability in how gaming time and mental health outcomes were measured across studies may affect the consistency and accuracy of the results.

## Conclusions

In conclusion, the relationship between screen time, particularly video gaming, and youth mental health is complex and requires further investigation. While excessive gaming is associated with increased risks of depression, self-harm, and suicidal ideation, it is essential to recognize that not all screen time has the same effects. Game content, social interactions, and gender differences significantly influence outcomes. More research is needed into the effects of video gaming on mental health, including distinguishing exacerbation of pre-existing conditions and differentiation of video game genres, for example. 

Parents, educators, and healthcare professionals must actively monitor and manage screen time to support the mental well-being of youth, promoting healthier gaming habits. Continued research is crucial to understanding these dynamics and developing effective interventions tailored to the diverse needs of children and adolescents.
